# EpiGRAPH: user-friendly software for statistical analysis and prediction of (epi)genomic data

**DOI:** 10.1186/gb-2009-10-2-r14

**Published:** 2009-02-10

**Authors:** Christoph Bock, Konstantin Halachev, Joachim Büch, Thomas Lengauer

**Affiliations:** 1 Max-Planck-Institut für Informatik, Campus E1.4, 66123 Saarbrücken, Germany

## Abstract

The EpiGRAPH web service  enables biologists to uncover hidden associations in vertebrate genome and epigenome datasets. Users can upload sets of genomic regions and EpiGRAPH will test multiple attributes (including DNA sequence, chromatin structure, epigenetic modifications and evolutionary conservation) for enrichment or depletion among these regions. Furthermore, EpiGRAPH learns to predictively identify similar genomic regions. This paper demonstrates EpiGRAPH's practical utility in a case study on monoallelic gene expression and describes its novel approach to reproducible bioinformatic analysis.

## Abstract 2

EpiGRAPH is a genome-scale data-mining software tool that enables users to identify epigenetic and gene regulatory features in large datasets of genomic regions.

## Rationale

EpiGRAPH addresses two tasks that are common in genome biology: discovering novel associations between a set of genomic regions with a specific biological role (for example, experimentally mapped enhancers, hotspots of epigenetic regulation or sites exhibiting disease-specific alterations) and the bulk of genome annotation data that are available from public databases; and assessing whether it is possible to predictively identify additional genomic regions with a similar role without the need for further wet-lab experiments.

The increasing relevance of analyzing sets of genomic regions arises from technical innovations such as tiling microarrays and next-generation sequencing [1-5], which can be used to scan the genome for specific types of regions (for example, transcription factor binding sites or cancer-specific genomic alterations). The resulting datasets are difficult to analyze with existing toolkits for genomic data mining - such as GSEA [6] and DAVID [7] - because most existing tools are gene-centric and cannot easily account for genomic regions that are located outside of (protein-coding) genes. In the absence of a suitable tool for statistical analysis and prediction of genomic region data, researchers have performed the necessary steps by hand, downloading relevant datasets from existing repositories and writing one-time-use scripts for data integration, statistical analysis and prediction (for example, [8-19]). Such manual analyses are time-consuming to perform, difficult to reproduce and require bioinformatic skills that are beyond the reach of most biologists. Hence, these studies support demand for a software toolkit that facilitates statistical analysis and prediction of region-based genome and epigenome data.

With the development of EpiGRAPH, we have pulled together our experiences and established workflows from several studies [10,20-23] and incorporated them into a powerful and easy-to-use web service. In the remainder of this paper, we sketch the basic concepts of EpiGRAPH, demonstrate its practical use and utility in a case study on monoallelic gene expression, and outline how the UCSC Genome Browser [24], Galaxy [25,26] and EpiGRAPH integrate into a comprehensive pipeline for (epi)genome analysis and prediction. Finally, the Methods section provides extensive bioinformatic background on EpiGRAPH's software architecture and describes how the software can be extended and customized. This paper is supplemented by a step-by-step, tutorial-style description of two example analyses [27] and by three tutorial videos that demonstrate EpiGRAPH 'in action' [28].

## Concept

EpiGRAPH is designed to facilitate complex bioinformatic analyses of genome and epigenome datasets. Such datasets frequently consist of sets of genomic regions that share certain properties, for example, being bound by a specific transcription factor or exhibiting characteristic patterns of evolutionary conservation. Typically, these genomic regions fall into opposing classes, for example, transcription factor bound versus unbound promoter regions or significantly conserved versus nonconserved regulatory elements. Even when this convenient situation does not emerge by default, it is straightforward and common practice to establish it artificially, by generating a randomized set of control regions to complement a given set of genomic regions. EpiGRAPH thus focuses on the analysis of sets of genomic regions that fall into two classes, which we denote as 'positives' (cases) and 'negatives' (controls).

**Figure 1:**
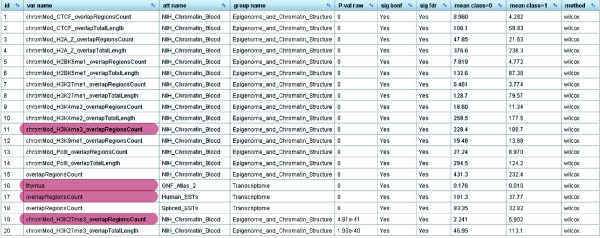
Results screenshot of EpiGRAPH's statistical analysis identifying significant differences between the promoter regions of monoallelically versus biallelically expressed genes. Comparing the promoter regions of monoallelically expressed genes (class = 1) with those of biallelically expressed genes (class = 0), EpiGRAPH's statistical analysis detects highly significant differences in terms of chromatin structure and transcriptional activity. *P*-values in this table are based on the nonparametric Wilcoxon rank-sum test ('method' column). Multiple hypothesis testing was accounted for with both the highly conservative Bonferroni method ('sig bonf' column) and the false discovery rate method ('sig fdr' column). A global significance threshold of 5% was used in both cases. Attributes highlighted in red are discussed in the main text. An explanation of attribute names is available from the EpiGRAPH website [29].

**Figure 2:**
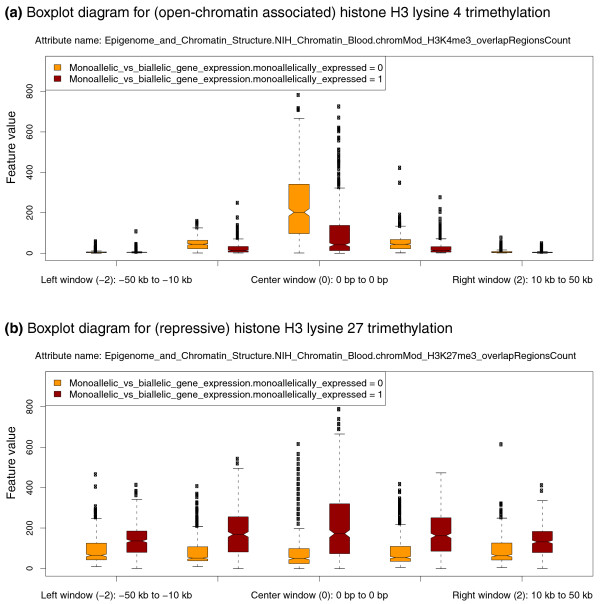
EpiGRAPH-generated diagrams highlighting differential histone modification patterns for the promoters of monoallelically versus biallelically expressed genes. This figure displays EpiGRAPH-generated boxplots comparing the promoter regions of genes exhibiting monoallelic (red boxplots) versus biallelic gene expression (yellow boxplots) with respect to their enrichment for two histone modifications, **(a) **H3 lysine 4 trimethylation and **(b) **H3 lysine 27 trimethylation. The y-axis plots the frequency of overlap with ChIP-seq tags [37], which is indicative of the strength of enrichment of the corresponding histone modification. Boxplots are in standard format (boxes show center quartiles, whiskers extend to the most extreme data point, which is no more than 1.5 times the interquartile range from the box) and outliers are shown as crosses.

**Figure 3:**
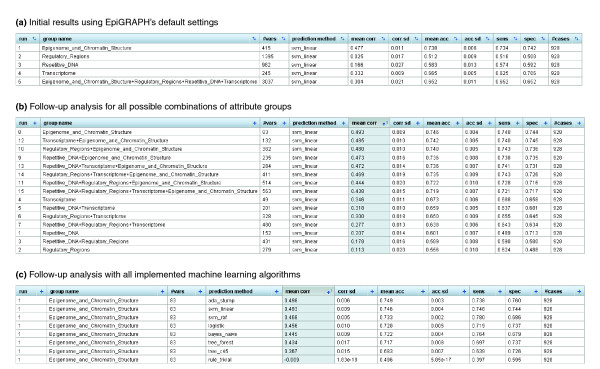
Results screenshots of EpiGRAPH's machine learning module predicting monoallelic gene expression. **(a-c) **These screenshots display the results of machine learning analyses comparing the promoter regions of monoallelically expressed genes (class = 1) with those of biallelically expressed genes (class = 0), each panel being based on different EpiGRAPH settings. The table values in the tables summarize the average performance of a linear support vector machine or alternative machine learning algorithms (c) that were trained and evaluated in ten repetitions of a tenfold cross-validation. Performance measures include mean correlation ('mean corr' column), prediction accuracy ('mean acc' column), sensitivity ('sens' column) and specificity ('spec' column). Additional columns display standard deviations observed among the repeated cross-validations with random partition assignment ('corr sd' and 'acc sd'), the number of variables in each attribute group ('#vars') and the total number of genomic regions included in the analysis ('#cases').

**Figure 4:**
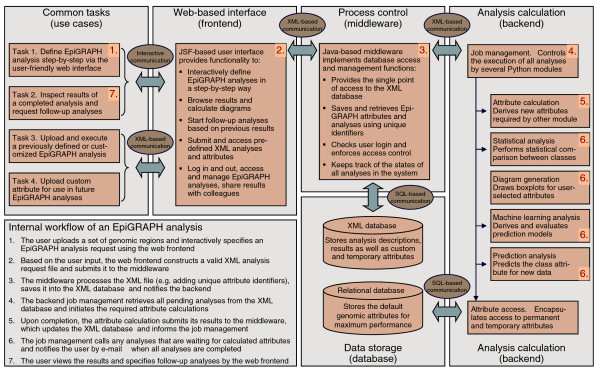
Outline of EpiGRAPH's software architecture. This figure displays a schematic overview of EpiGRAPH's software components, and it describes their interaction in a typical analysis workflow. The red numbers indicate the key component(s) for each step of the workflow description outlined in the bottom left of the figure. JSF, Java Server Faces (which is a Java-based web application framework).

EpiGRAPH provides four analytical modules (see Figures 1, 2, 3 for screenshots of illustrative results and Figure 4 for an overview of EpiGRAPH's software architecture). The statistical analysis module identifies attributes that differ significantly between the sets of positives and negatives, based on an attribute database comprising a broad range of genome and epigenome datasets. The diagram generation module draws boxplots that visualize the distribution of a selected attribute among the sets of positives versus negatives. The machine learning analysis module evaluates how well prediction algorithms - such as support vector machines - can discriminate between positives and negatives in the input dataset, based on different combinations of (epi)genomic attributes from the database. The prediction analysis module predicts whether a genomic region that is not contained in the input dataset belongs to the set of positives or negatives, thus exploiting any correlations detected by the machine learning analysis module for the prediction of new data.

Typical EpiGRAPH analyses follow a defined workflow. The starting point is a dataset of genomic regions, which the user may have obtained through wet-lab analysis (for example, ChIP-seq analysis of transcription factor binding) or bioinformatic calculations (for example, computational screening for regions that are under evolutionary constraint). This dataset is uploaded to the EpiGRAPH web service as a table of genomic regions with separate columns for chromosome name, start position, end position, and a binary class value specifying for each region whether it belongs to the positives or negatives. (When no class value is provided, EpiGRAPH regards all genomic regions of the input dataset as positives and assists the user with calculating a set of random control regions to be used as negatives.) Next, EpiGRAPH calculates a large number of potentially relevant attributes for each genomic region in the input dataset. Most of these attributes represent overlap frequencies or score values, quantifying the co-localization of the genomic regions in the input dataset with publicly available annotation data for the respective genome. Upon completion of the attribute calculation (which can take several hours or even days when the input dataset is large), EpiGRAPH's statistical and machine learning modules test for significant differences between the positives and negatives in the input dataset and perform an initial assessment of whether or not these differences are sufficient for bioinformatic prediction. Based on an inspection of these results, the user can request follow-up analyses utilizing the pre-calculated data. In particular, the diagram generation module can be used to visualize interesting differences between positives and negatives as detected by the statistical analysis, and the prediction analysis module lets the user predict the class value of new genomic regions - for example, in order to extrapolate experimental data to regions that were not covered by wet-lab experiments.

The key to EpiGRAPH's practical utility is its database, for which we collected a large number of attributes that are likely to play a role in genome function and epigenetic regulation. For the most thoroughly annotated human genome, EpiGRAPH currently includes almost a thousand attributes (see Table 1 for an overview and the attribute documentation website [29] for details). These attributes fall into ten groups: DNA sequence; DNA structure; repetitive DNA; chromosome organization; evolutionary history; population variation; genes; regulatory regions; transcriptome; and epigenome and chromatin structure. EpiGRAPH also incorporates the genomes of chimp, mouse and chicken (with slightly lower numbers of attributes) and can easily be extended to support genomes of other species. In addition to using EpiGRAPH's default attributes, researchers can upload their own datasets and incorporate them as custom attributes in subsequent analyses. This is particularly useful because problem-relevant experimental data - such as chromatin structure data for the cell type of interest - often boost EpiGRAPH's prediction accuracy.

**Table 1:** List of default attributes included in EpiGRAPH

	Total number of attributes					
		
Attribute groups	*hg18*	*hg17*	*mm9*	*panTro2*	*galGal3*	Attributes (examples)
DNA sequence	178	178	178	178	178	Frequency of 'TATA' pattern, cytosine content, CpG frequency
DNA structure	21	21	21	21	21	Predicted DNA helix twist, predicted solvent accessibility
Repetitive DNA	95	95	91	94	94	Overlap with Alu elements, LINEs and tandem repeats
Chromosome organization	18	29	15	-	-	Overlap with chromosomal bands and isochors
Evolutionary history	94	101	-	-	86	Overlap with evolutionarily conserved regions
Population variation	75	75	-	-	-	SNP density and overlap with specific SNP types (for example, non-synonymous exonic or splice site)
Genes	37	60	20	10	10	Overlap with annotated genes, pseudogenes and predicted microRNA genes
Regulatory regions	249	259	5	5	5	Overlap with CpG islands and predicted transcription factor binding sites
Transcriptome	49	65	9	9	9	Overlap with ESTs and mRNA sequences
Epigenome and chromatin structure	80	17	114	-	-	Overlap with ChIP-seq tags indicating enrichment for specific histone modifications

Sum	896	900	453	317	403	

This table summarizes the collection of default attributes that are currently included in EpiGRAPH. Due to different degrees of annotation, the numbers differ between the genomes of human (*hg18 *and *hg17*), mouse (*mm9*), chimp (*panTro2*) and chicken (*galGal3*). EST, expressed sequence tag; SNP, single nucleotide polymorphism.

## Application

The best starting point for getting acquainted with the practical use of EpiGRAPH are the tutorial videos [28] and the step-by-step guide [27], which is available online. In the following case study, we take a slightly more high-level view, focusing on how to plan and interpret an EpiGRAPH analysis and highlighting potential sources of misinterpretation. All raw data, settings and results of this case study are available online [30], and readers are encouraged to download the analysis description file, upload it into their own EpiGRAPH accounts, reproduce the results and perform follow-up analyses.

Monoallelic gene expression - the focus of our case study - is a common phenomenon in vertebrate genomes. While the majority of human genes are expressed from both alleles, a sizable proportion is expressed exclusively from a single allele, with important biological consequences. Genomic imprinting - that is, parent-specific monoallelic gene expression - plays a critical role in normal development and gives rise to non-Mendelian patterns of inheritance [31]. X-chromosome inactivation leads to mitotically heritable silencing of the surplus X chromosome in females [32]. And random monoallelic gene expression, which is common among odorant receptor genes and immune-system related genes, increases the phenotypic diversity among clonal cells [33].

In an attempt to identify potential determinants of monoallelic gene expression, several bioinformatic studies compared DNA sequence properties of monoallelically versus biallelically expressed genes. These studies reproducibly found enrichment of long interspersed nuclear element (LINE) repeats and depletion of short interspersed nuclear element (SINE) repeats to be associated with monoallelic gene expression [8,34-36]. Encouraged by this finding, attempts have been made to predict - based on the genomic DNA sequence - which genes are subject to imprinting and X-chromosome inactivation [16,17,19]. However, the conclusiveness of these prior studies is somewhat diminished by the fact that most of them relied on small gene lists curated from the literature and that none took epigenome data into account.

Here, we revisit the relationship between DNA characteristics and monoallelic gene expression based on genome-scale datasets, including a recent assessment of monoallelic versus biallelic gene expression for about 4,000 genes in human lymphoblastic cells [33] and extensive epigenome maps of human T-cell lymphocytes [37]. To start with, we obtain a list of monoallelically and biallelically expressed genes from the supplementary material of the corresponding paper [33], and we map these to a non-redundant set of RefSeq gene promoters (this step is performed using Galaxy [38]). As the result, we obtain a total of 464 positives (monoallelically expressed genes) as well as a substantially longer list of negatives (biallelically expressed genes), from which we randomly select 464 genes to match the number of positives. Random down-sampling of the set of negatives is performed in order to limit bias toward predicting the majority class, which is a common issue in machine learning. In general, we recommend that the number of positives should never exceed twice the number of negatives, and vice versa. EpiGRAPH automatically enforces this upper limit for the class imbalance, unless the user deselects the corresponding option.

Before we can submit our dataset to EpiGRAPH, we have to decide exactly which regions we want to analyze, that is, whether we expect DNA signals relating to monoallelic gene expression distributed throughout the gene or preferentially located in specific regions, such as promoters, exons or introns. Since monoallelic gene expression appears to be controlled by the transcriptional machinery, we believe that promoter regions have the highest probability of containing relevant regulatory elements. For the purpose of this analysis, we define the putative promoter region as the sequence window ranging from 1,250 bp upstream to 250 bp downstream of the annotated transcription start site. We calculate the corresponding region of interest for each gene in our dataset, giving rise to the input file that can be uploaded to EpiGRAPH. However, as we cannot exclude that important regulatory elements might be located further upstream or downstream, we activate EpiGRAPH's option to cover four additional sequence windows ranging from -50 kilobases to +50 kilobases around the region of interest.

Next, we have to decide which groups of attributes from EpiGRAPH's database to include in our analysis. While it is always possible to perform hypothesis-free screening by selecting all default attributes, focusing the analysis only on promising attribute groups can significantly increase statistical power and also decreases computation time. Based on prior knowledge, we choose four attribute groups that are likely to be related to monoallelic gene expression, namely 'repetitive DNA', 'regulatory regions', 'transcriptome', and 'epigenome and chromatin structure'.

Having made all relevant decisions, we can now start the analysis, log out of the web service and wait for EpiGRAPH to perform the necessary calculations. Assuming that email notification has been enabled, EpiGRAPH will inform us as soon as it has completed an initial analysis. At that point, we can log into the web service again, review the results and define follow-up analyses.

Our inspection of the results starts with the statistical analysis table (Figure 1). This table summarizes pairwise statistical comparisons between positives and negatives, which were performed for each attribute using Wilcoxon's rank-sum test (for numerical attributes) and Fisher's exact test (for categorical attributes). Focusing on the 1.5 kilobase core promoter region (the main window of our analysis), a total of 72 out of 563 attributes differ significantly between monoallelically and biallelically expressed genes, at a false discovery rate of 5%. Furthermore, similar but weaker differences are observed for four additional sequence windows upstream and downstream of the promoter region (data not shown), indicating that the contrasting genomic properties of monoallelically versus biallelically expressed genes are strong for the core promoter, but also present in a wider genomic region surrounding the genes.

In their core promoter regions, biallelically expressed genes exhibit, on average, twice the amount of histone H3 lysine 4 trimethylation (which is indicative of open chromatin) as the promoters of monoallelically expressed genes. Conversely, the latter are almost threefold enriched in terms of repressive histone H3 lysine 27 trimethylation. Consistent with the interpretation that promoters of monoallelically expressed genes generally exhibit a more repressed chromatin state than their biallelic counterparts, we also observe significant under-representation of their associated transcripts in expressed sequence tag (EST) libraries and decreased expression according to microarray data (Figure 1). Interestingly, out of the 28 tissues covered by EpiGRAPH, the difference in gene expression is most significant for thymus, consistent with the fact that monoallelic gene expression is prominent among genes related to the immune system.

To illustrate the distinct chromatin structure at the core promoters of monoallelically versus biallelically expressed genes, we select H3 lysine 4 trimethylation and H3 lysine 27 trimethylation for visualization using EpiGRAPH's diagram generation module (Figure 2). Boxplots confirm that the differences are not only significant, but also substantial in quantitative terms. This confirmation is an important first step toward establishing the biological relevance of our finding, given that even minor and biologically irrelevant differences can become highly significant when sample sizes are large. In general, to demonstrate both significance and strength of an observed difference, we recommend that EpiGRAPH users should report not only *P*-values, but also the corresponding boxplot diagrams or at least separate mean values for the sets of positives and negatives.

Further support for a strong association between (repressive) chromatin structure and monoallelic gene expression comes from EpiGRAPH's machine learning analysis. Based on the values of 83 chromatin-related attributes measured across the core promoter regions and four adjacent windows (415 variables in total), EpiGRAPH could predict with an accuracy of 73.8% (sensitivity, 73.4%; specificity, 74.2%; correlation, 0.47) whether a gene is monoallelically or biallelically expressed (Figure 3a). Substantially lower prediction performance was observed for the other attribute groups, namely repetitive DNA (accuracy, 58.3%; correlation, 0.17), regulatory regions (accuracy, 51.2%; correlation, 0.03) and the transcriptome (accuracy, 66.5%; correlation, 0.33). We thus conclude that attributes relating to epigenome and chromatin structure are among the most significant predictors of monoallelic gene expression. Importantly, all measures of prediction performance reported by EpiGRAPH are calculated exclusively based on test set results in a cross-validation design, thereby minimizing the risk of overtraining and irreproducibly optimistic performance evaluations that is inherent in the use of machine learning methods [39].

Due to the complex structure of mammalian genomes, the attribute groups included in our analysis are not statistically independent. On the contrary, strong biological interdependencies exist between different attribute groups - for example, between chromatin structure and the transcriptome (open chromatin structure facilitates transcription), between regulatory regions and repetitive DNA (regulatory regions are preferentially located in non-repetitive regions), and between repetitive DNA and chromatin structure (repetitive regions most commonly exhibit repressive chromatin structure). Therefore, the predictiveness of some attribute groups included in our analysis could be indirect and mediated by their correlation with other, more predictive attributes. EpiGRAPH helps us better understand such relationships by measuring whether any combination of two or more attribute groups gives rise to higher prediction performance than each attribute group on its own right (which indicates that all attribute groups contribute to the overall prediction performance) or whether a single attribute group dominates the other attribute groups (in which case the other attribute groups are likely to 'borrow' predictiveness from the former, rather than being independently predictive). To perform such an analysis, we restart the machine learning analysis with custom settings, requesting EpiGRAPH to account for all possible combinations of attribute groups while focusing on the putative promoter regions (that is, ignoring the four additional sequence windows upstream and downstream). The results table lists prediction performance separately for linear support vectors trained on each of the 15 possible combinations of attribute groups (Figure 3b). These data clearly indicate that a single attribute group - epigenome and chromatin structure - is more predictive than all others. In fact, there is no evidence of complementarity for any combination of attribute groups (that is, no set of attribute groups outperforms the single highest-scoring attribute group contained in the set). In the light of these results, it seems unlikely that repetitive elements are directly causal for monoallelic gene expression, at least on a genomic scale. Rather, the predictiveness of specific repetitive elements observed in prior studies as well as in this analysis appears to be largely due to the fact that certain types of repeats (such as LINEs) are enriched in regions that exhibit repressive chromatin structure, while other types of repeats (such as SINEs) are depleted in such regions.

In a final step, we want to use EpiGRAPH to predict for all genes in the human genome whether their tendency is toward monoallelic or biallelic gene expression. To that end, we first verify that a linear support vector machine (EpiGRAPH's default prediction algorithm) indeed provides competitive prediction performance when compared to other machine learning algorithms. Such benchmarking is achieved by restarting the machine learning analysis with custom settings and selecting all available machine learning algorithms for inclusion (Figure 3c). EpiGRAPH's cross-validation results indicate that linear support vector machines perform on par with the best method, an ensemble learning algorithm (AdaBoost on tree stumps). We thus conclude that a linear support vector machine trained on epigenome and chromatin structure data provides a suitable setup for genome-wide prediction of monoallelic gene expression. Next, we obtain a list of RefSeq-annotated genes from the UCSC Genome Browser, calculate the 1.5 kilobase promoter regions for all genes and submit this dataset to EpiGRAPH's prediction analysis. Upon submission of the analysis, EpiGRAPH starts to calculate the relevant attributes and predicts the expression status of all 25,419 RefSeq-annotated genes in the human genome. The results - which are available online [30] - provide a first genome-wide prediction of monoallelic gene expression in the human genome. Although the accuracy of our predictions is far from perfect (Figure 3c) and further experimental analysis is clearly warranted, these predictions could be useful for identifying new candidate genes that contribute to the many biological roles of monoallelic gene expression.

In summary, this case study illustrates how EpiGRAPH can be applied to analyzing a genomic feature of interest (in this case, monoallelic gene expression) in the context of publicly available genome annotations and epigenome data. Two main conclusions emerge from our analysis. First, monoallelically expressed genes exhibit a substantially more repressed chromatin structure in their promoter regions than biallelically expressed genes. This observation is consistent with a model in which monoallelic gene expression is the direct consequence of opposing chromatin states at the two alleles of a gene within a diploid cell. Indeed, Wen *et al*. [40] recently showed that an experimental search for genomic regions that exhibit activating as well as repressive chromatin marks can identify monoallelically expressed genes. Second, chromatin structure clearly emerges as the strongest predictor of monoallelic gene expression, outperforming attributes such as the overall level of gene expression or the enrichment/depletion of specific types of repeats and regulatory regions. In fact, none of the other attribute groups included in our analysis could increase prediction performance after chromatin structure had been accounted for. This observation is not necessarily in contradiction with an (indirectly) causal model in which local enrichment of LINEs fosters repressive chromatin structure, which in turn facilitates random silencing of a single allele. However, the weak predictiveness of attributes relating to repetitive DNA suggests that such a model omits important additional drivers of monoallelic gene expression.

## Integration

**Figure 5:**
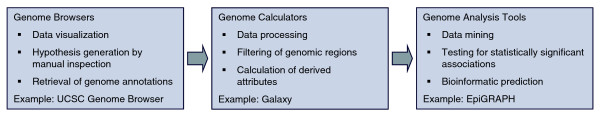
Workflow for web-based analysis of large genome and epigenome datasets. This figure outlines a workflow for the analysis of genome and epigenome data using publicly available web services. Initially, the user uploads a newly generated dataset into a genome browser, which visualizes the data and facilitates hypothesis generation by manual inspection (left box). Next, data can be processed with a genome calculator such as Galaxy, in order to extract interesting regions for in-depth analysis (center box). Finally, genome analysis tools such as EpiGRAPH facilitate the search for significant associations with genome annotation data and enable bioinformatic prediction of genomic regions with similar characteristics as the input dataset (right box).

EpiGRAPH integrates well with existing bioinformatics resources and infrastructure. It can be regarded as part of a three-step data analysis pipeline involving genome browsers, genome calculators and tools for genome data analysis (Figure 5). First, researchers typically start the analysis of new genome-scale datasets by uploading pre-processed and quality-controlled data into a genome browser, which facilitates data visualization and manual inspection. The UCSC Genome Browser [24] is popular for this task, due to the ease with which custom data tracks can be displayed alongside public genome annotations, and Ensembl is an alternative option [41]. Second, based on initial observations, it is usually necessary to pick a subset of genomic regions for further analysis - for example, all promoter regions that are bound by a specific transcription factor. The Galaxy web service [25,26] implements a wide range of calculations and filtering methods that facilitate the selection of biologically interesting regions for further analysis. Finally, it is often desirable to perform statistical analysis and data mining on the potentially large set of interesting regions in order to discover, test and interpret correlations with other genomic data. For this step, a comprehensive and easy-to-use toolkit has been lacking. We developed EpiGRAPH to fill this gap, thereby enabling biologists to perform advanced bioinformatic analysis and prediction with little need for bioinformatic support. We demonstrate the interplay of UCSC Genome Browser, Galaxy and EpiGRAPH in a case study focusing on the (epi)genomic characteristics of highly polymorphic promoter regions in the human genome [27,28].

In the future, we anticipate that the three layers of genome browsing, calculation and analysis tools will increasingly merge into a single application, for which 'statistical genome browser' might be an appropriate term. To that end, it will be neither necessary nor beneficial to integrate all functionality and underlying databases into a single monolithic tool. Instead, a distributed network of interoperable web services for genome analysis is likely to emerge. Genome browsers could act as single points of entry, from which the user initiates a complex analysis. The analysis is then split into separate subtasks, encoded in an XML-based analysis description language (such as the XML genomic relationship analysis format (X-GRAF) prototyped in EpiGRAPH) and distributed over the Internet to calculation servers at which all relevant datasets and software components for a specific type of analysis are available. Finally, the decentrally calculated results are merged and displayed to the user at the central genome browser front-end. EpiGRAPH was developed with this scenario in mind and prototypes software paradigms required for distributed genome analysis by concerted action of specialized tools.

## Conclusion

The EpiGRAPH web service enables biologists to perform complex bioinformatic analyses online - without having to learn a programming language or to download and manually process large datasets. Compared to related tools such as Galaxy [25,26] and Taverna [42,43], its main emphasis lies in exploratory statistical analysis, hypothesis generation and bioinformatic prediction, based on large datasets of genomic regions. EpiGRAPH facilitates reproducibility and data sharing by encoding all analyses in standardized analysis description files that can be re-run by other users. We highlighted EpiGRAPH's utility by a case study on monoallelic gene expression, and we provide extensive additional material online (including tutorial videos and a step-by-step guide [27,28]).

## Methods

### EpiGRAPH's software architecture and analysis workflow

The key design decision underlying EpiGRAPH's software architecture is to store each EpiGRAPH analysis in a single XML file. This XML file contains not only a detailed specification of the analysis and its supplementary attributes, but also its current processing status and, upon completion, its results. All XML files processed by EpiGRAPH conform to the standardized X-GRAF format (discussed in more detail below) and are stored in an XML database.

EpiGRAPH's XML-based, analysis-centric design offers a number of advantages over alternative architectures, including reproducibility, parallel processing and interoperability and error checking. Reproducibility: all information relevant to an analysis, including its specifications and results, are bundled in a single file, which provides a complete documentation of the analysis. The same analysis can be rerun at any time simply by uploading its XML file back to the EpiGRAPH web service. Parallel processing: because the different analysis modules operate on different parts of the XML tree, they can work in parallel without generating write-write conflicts. Interoperability and error checking: the use of XML files facilitates data exchange with other software systems, and the X-GRAF format provides error checking when XML files are constructed manually or exchanged between different software systems.

Internally, the EpiGRAPH web service consists of three software components and two logical databases (Figure 4). The web-based front-end provides user-friendly access to EpiGRAPH's functionality over the internet. The front 0 end is implemented in Java [44], utilizing the JavaServer Faces framework for its user interface and Java servlets as well as JavaServer Pages for operating as a web application. The process control middleware provides a single point of access to the analyses and custom attributes stored in the XML database, and it enforces compliance with the X-GRAF XML format. The middleware is implemented as a Java servlet and makes its services available via XML-RPC [45]. The analysis calculation back-end performs all attribute calculations and bioinformatic analyses required to execute an EpiGRAPH analysis request. It submits its results to the middleware, which stores them in the XML database. The back-end is implemented in Python [46], using the R package [47] for statistical analysis and diagram generation, and the Weka package [48] for machine learning and prediction analysis. The relational database stores EpiGRAPH's default attributes. Oracle Database 11 g [49] is used with pre-calculated indices in order to achieve high-performance database retrieval. The XML database provides central storage of all XML files and enables parallelized access to the XML files as a whole as well as to specific subnodes. EpiGRAPH makes use of Oracle XML DB [50], which is an XML database extension of the Oracle database. Technically, Oracle XML DB decomposes all XML files into relational database tables, based on the X-GRAF schema definition and object-relational mapping. Hence, while the relational database and the XML database behind EpiGRAPH are logically distinct and used for different types of data (default attributes versus analysis requests and custom attributes), both types of data are ultimately stored in the same database management system.

Importantly, the choice of technologies for each component reflects the specific requirements of the tasks they perform. The front-end has to present a user-friendly interface in a variety of web browsers, which is facilitated by a web application framework such as JavaServer Faces. The middleware makes connections with the XML database and performs extensive XML processing; hence, Java, with its well-established libraries for Oracle XML DB access [50], StAX [51] and JAXB processing [52], is an appropriate choice. The back-end implements most of EpiGRAPH's application logic and is likely to be extended by other researchers, therefore Python [46] was selected due to its proven track record for fast and robust software engineering in scientific applications, its platform independence and its wide acceptance within the bioinformatics community.

The internal workflow of an EpiGRAPH analysis is depicted in Figure 4, illustrating how the different components interact when fulfilling an EpiGRAPH analysis request.

### Genomes, annotations and attributes included in EpiGRAPH

EpiGRAPH currently supports five genome assemblies from four species: *hg18*, the latest assembly of the human genome (NCBI36.1); *hg17*, the genome assembly used for the ENCODE project pilot phase (NCBI35); *mm9*, the latest assembly of the mouse genome (NCBI37); *panTro2*, the latest assembly of the chimp genome; and *galGal3*, the latest assembly of the chicken genome. For each of these genomes, we manually selected a large number of genomic attributes that are likely to be predictive of interesting genomic phenomena (see Table 1 for an overview and the attribute documentation website [29] for details). When calculated for a specific genomic region, most of these attributes take the form of overlap frequencies (for example, how many exons overlap with the genomic region?), overlap lengths (for example, how many base-pairs of exonic DNA overlap with the genomic region?) or DNA sequence pattern frequencies (for example, how many times does the pattern 'TATA' appear in the genomic region?). All of these attributes are standardized to a default region size of one kilobase in order to be comparable between genomic regions of different size. In addition, EpiGRAPH uses score attributes, which are averaged across all overlapping regions of a specific type (for example, what is the average exon number of all genes overlapping with the genomic region?), and category attributes, which split up an attribute into subattributes (for example, how many synonymous versus non-synonymous single nucleotide polymorphisms overlap with the genomic region?).

The datasets underlying most of these attributes were collected from annotation tracks of the UCSC Genome Browser [24], using an automated data retrieval pipeline. In addition, published genomic datasets that appear to be of particular interest are imported into the database on a regular basis. Currently, this includes data on histone modifications [37], DNA methylation [53,54], regulatory CpG islands [20], DNA helix structure [55], DNA solvent accessibility [56], tissue-specific gene expression [57], isochores [58] and transcription initiation events [59]. Finally, users can upload custom datasets into the database, making them available for inclusion in further analyses by the same user.

### Attribute calculation

The basic functionality of EpiGRAPH's attribute calculation module is to calculate a large number of genomic attributes (such as frequency and length of overlap with EpiGRAPH's default attributes) for any set of genomic regions submitted to the web service. This step is a prerequisite for all further analyses, and it is typically the most computationally intensive and time-consuming part of an EpiGRAPH analysis. The attribute calculation makes extensive use of multithreading in order to increase performance.

Beyond its core task of deriving hundreds or even thousands of different attribute values for each genomic region in the input dataset, the attribute calculation module provides three additional features that increase its utility as a general genome calculator. First, the user can define derived attributes, thus augmenting genomic attributes that are already contained in the database (for example, deriving a set of putative promoter regions from a gene attribute). Second, random control regions can be calculated such that they match a given set of genomic regions in terms of chromosome and length distribution, GC content, repeat content and/or exon overlap. Technically, this is achieved by repeatedly sampling random genomic regions of a given length from a specific chromosome and retaining a region only if its GC content, repeat content and/or exon overlap are within a user-specified interval around the corresponding value of the source region. Third, attributes can be calculated not only for the genomic regions provided in the input dataset, but also for fixed sequence windows left and right of these regions, in order to capture significant differences in the upstream or downstream neighborhood of a given set of genomic regions. All results calculated by the attribute calculation module can be used as the basis for further EpiGRAPH analyses or downloaded in tab-separated value format for analysis outside EpiGRAPH.

### Statistical analysis and diagram generation

Two of EpiGRAPH's four analytical modules - statistical analysis and diagram generation - help the user identify individual attributes that differ between two sets of genomic regions, which we denote as 'positives' and 'negatives'. The statistical analysis module calculates pairwise statistical tests between the positives and negatives separately for each genomic attribute. The nonparametric Wilcoxon rank-sum test is used for numeric attributes and Fisher's exact test is used for discrete attributes. *P*-values are adjusted for multiple testing by the highly conservative Bonferroni method, which controls the family-wise error rate, and by a more recent and usually preferred method that controls the false discovery rate [60]. While EpiGRAPH applies an overall significance threshold of 5% by default, the user is free to select different values. If multiple windows around the genomic regions of interest are taken into account and tested simultaneously, the user can specify weights to control how the *P*-value threshold is distributed when testing for significant attributes in each of these windows. A typical choice is to use a relatively high *P*-value of, say, 3% for the central window (that is, the regions provided by the input dataset), while distributing the remaining 2% equally among the upstream and downstream windows. This way, the additional testing for strong effects in the upstream and downstream neighborhoods comes at the cost of only a limited decrease in statistical power for the genomic regions of interest.

While the statistical analysis module focuses on the question of whether or not a specific attribute differs significantly between the sets of positives and negatives, the diagram generation module can help assess the effect size, that is, the quantitative difference between positives and negatives. For any selected attribute, this module derives boxplots contrasting the attribute's distribution among the positives with that among the negatives.

### Machine learning analysis and prediction analysis

In contrast to the statistical analysis module, which focuses on individual attributes, the machine learning analysis module assesses how well attribute groups collectively differentiate between the sets of positives and negatives. We treat this question as a machine learning task, predicting for each genomic region whether it is likely to belong to the set of positives or to the set of negatives and interpreting the prediction performance achieved for a specific attribute group as a measure of how well this group discriminates between positives and negatives.

Technically, a machine learning algorithm (for example, a support vector machine) is repeatedly trained and tested on partitions of the training dataset following a four-step procedure (all parameters mentioned below are default values and can be changed by the user). First, if the set of positives contains more than twice as many genomic regions as the set of negatives (or vice versa), the larger set is randomly downsampled such that the class imbalance never exceeds 67% versus 33%, thus limiting potential prediction bias toward the majority class. Second, using tenfold cross-validation, the machine learning algorithm is repeatedly trained on 90% of the genomic regions and tested on the remaining 10%. Third, cross-validation is repeated ten times with random partition assignments. Fourth, the overall prediction performance is measured by the correlation coefficient between the predictions and the correct values on the cross-validation test sets, as well as by the corresponding values for percent accuracy, sensitivity and specificity, averaged over all cross-validation runs.

During prediction analysis, a machine learning algorithm is trained as described above, but now on a bootstrapped sample drawn from the entire training dataset (downsampling is used if necessary to enforce a maximum class imbalance of 67% versus 33%). The trained prediction model is then applied to predict the likelihood of belonging to the set of positives for all genomic regions in a user-supplied set of target regions. The resulting quantitative prediction for each region can assume values between zero and one, with a value of zero corresponding to a high-confidence negative prediction, a value of 0.5 to a borderline case, and a value of one to a high-confidence positive prediction. This process is repeated ten times with different bootstrapped samples in order to obtain an additional criterion for the reliability of the predictions. Finally, the consensus prediction, the mean confidence value and the standard deviation of the confidence values are calculated for each genomic region and each prediction setup.

For both machine learning analysis and prediction analysis, EpiGRAPH currently supports the use of seven different machine learning methods/configurations: support vector machine with linear kernel; support vector machine with RBF kernel; AdaBoost on tree stumps; logistic regression; random forest; C4.5 tree generator; and naïve Bayes. All of these are implemented using functions from the Weka package [48] with default parameters. For comparison and to give a baseline for the expected accuracy, we also include a trivial algorithm that always predicts the majority class.

### X-GRAF format

Throughout EpiGRAPH's workflow (Figure 4), analyses and custom attributes are stored in XML files. In order to standardize the format of these XML files and to facilitate interoperability between the front-end, middleware and back-end components, we defined the X-GRAF format. X-GRAF consists of an XML schema, against which any X-GRAF-compatible XML file has to validate in order to be regarded as syntactically correct, and a set of rules that describe the semantic interpretation of X-GRAF-compliant XML files (detailed documentation is available online [61]). X-GRAF-compatible XML files can incorporate two major subtrees, 'attribute definition' and 'analysis' (an illustration is available online [62]). The attribute definition section keeps track of genomic attributes, which are organized in attribute groups and can be defined by embedded tab-separated tables or by referring to external data sources (such as a database or a URL). The analysis section documents all analysis steps, including attribute calculation, statistical analysis, diagram generation, machine learning analysis and prediction analysis. Each of these subsections comprises an analysis configuration (a description of what is to be calculated), analysis tracking information (for example, submission data, current state and error messages) and the results of the analysis (in the form of tables and diagrams that are directly embedded in the XML file).

Although X-GRAF was created for EpiGRAPH, it is designed with additional applications in mind. Being both formalized and sufficiently easy to understand, X-GRAF may provide a suitable basis for analysis specification, results documentation and data exchange of future genome analysis tools and statistical genome browsers.

### Adapting and extending EpiGRAPH

EpiGRAPH provides multiple options for customization, adaptation and extension, which are outlined below in increasing order of complexity and power.

First, it is possible to use EpiGRAPH for attribute calculation only, thus profiting from EpiGRAPH's large and carefully selected set of default attributes, while performing follow-up analyses offline (for example, with the R statistics package). To that end, the user performs a normal EpiGRAPH analysis and presses the 'Download Data Table' button on the results page to obtain a tab-separated data file that contains all attribute values for all genomic regions in the input dataset.

Second, the user can add custom genomic attributes to EpiGRAPH, using the 'Upload Custom Attribute Dataset' button on the overview page. A new custom attribute can be defined in three ways: by uploading a set of genomic regions; by specifying how the attribute can be calculated from other attributes that are already present in the database (for example, filtering rows that match a specific condition or defining additional columns); and by deriving a randomized control attribute that matches an existing attribute in terms of its GC content, repeat content and/or exon overlap. Custom attributes can be included in EpiGRAPH analyses in the same way as the default attributes, but they are exclusively accessible to the user who created them.

Third, the user can specify advanced analysis requests and attribute calculations directly in EpiGRAPH's internal X-GRAF format. Any XML file that adheres to the X-GRAF format can be uploaded through the 'Execute Analysis Based on Existing XML File' button, bypassing the interactive 'Define New Analysis' pages. This can be useful for several reasons: when running the same analysis on different datasets, it is often convenient to design the analysis once using the web front-end, then download its specifications in X-GRAF format and use a text editor or a custom script to produce separate versions for each dataset; sharing X-GRAF files with other researchers (for example, by inclusion in the supplementary material of a paper) will enable them to reproduce the analysis by simply submitting the X-GRAF files back to the EpiGRAPH web service, thus contributing to reproducible research [63]; and some of the more advanced features (for example, calculated attributes with multiple new columns) are supported by the calculation engine but cannot be specified easily using the web front-end.

Fourth, the user can download a 'light' version of the EpiGRAPH calculation engine for local installation, which runs on any computer with recent versions of Python [46], the R statistics package [47] and the Weka data mining package [48], after a few additional libraries have been installed. The 'light' version (source code available online [64]) is particularly useful for researchers developing new bioinformatic methods for genome analysis, such as new flavors of the statistical analysis, diagram generation, machine learning analysis and prediction analysis, but who do not want to spend their time writing code for attribute calculation. The main disadvantage of the 'light' version is that in the absence of a relational database all genomic attributes have to be stored in flat files. However, the 'light' version is code-compatible with the full version of EpiGRAPH. Hence it is possible to develop and test new modules using the 'light' version and to incorporate the completed modules into the EpiGRAPH web service.

Fifth, the user can obtain and install the full version of EpiGRAPH (release package and source code available on request), which includes the process control middleware and the web front-end components as well as a version of the calculation engine that provides full database support. While running a full-blown EpiGRAPH server locally is a non-trivial task and requires both a Java application server (for example, Apache Tomcat) and an Oracle 11 g database server [49], this setting gives the user full flexibility for customizing EpiGRAPH and a powerful infrastructure for genome analysis.

## Abbreviations

LINE: long interspersed nuclear element; SINE: short interspersed nuclear element; X-GRAF: XML genomic relationship analysis format.

## Competing interests

The authors declare that they have no competing interests.

## Authors' contributions

CB initiated the project, conceptualized the software, implemented the front-end, middleware and database components as well as an early back-end prototype, performed the case study and drafted the paper. KH designed and implemented a substantially enhanced version of the back-end, performed extensive testing and contributed important ideas to all aspects of the project. JB set up and maintained the technical infrastructure. All authors provided relevant input at different stages of the project and contributed to the writing of the paper.
